# UNIQUE MICROBIOME-PROTEOME SIGNATURES OF SALIVA IN PREDICTION OF COGNITIVE DECLINE IN OLDER ADULTS OF MIAGB COHORT

**DOI:** 10.1093/geroni/igad104.2994

**Published:** 2023-12-21

**Authors:** Diptaraj Chaudhari, Shalini Jain, Corinne Labyak, Adam Golden, Mia GB, Peter Holland, Michal Masternak, Hariom Yadav

**Affiliations:** University of South Florida, Tampa, Florida, United States; University of South Florida, Tampa, Florida, United States; University of North Florida, Jacksonville, Florida, United States; University of Central Florida College of Medicine, Orlando, Florida, United States; University of South Florida, Tampa, Florida, United States; Schmidt College of Medicine, Boca Raton, Florida, United States; University of Central Florida College of Medicine, Orlando, Florida, United States; University of South Florida, Tampa, Florida, United States

## Abstract

The prevalence of age-related cognitive decline and dementia is increasing in older adults, with limited prevention and therapeutic options, increasing tremendous burden on healthcare system. Alzheimer’s disease (AD) is the most common age-related cognitive disorder, for which no treatment is available when its pathology is fully developed. Although current FDA-approved therapies are proven to be effective in delaying AD progression, however, inexpensive and non-invasive markers for routine laboratory examination are poorly developed. Studies indicate that poor oral health is associated with a higher risk of dementia via microbiome alteration. In this pilot study, we explored the importance of the multiomics analysis in discriminating cognitively healthy from mild cognitive impaired and dementia by comparing the microbiome and proteomic profiles of saliva from controls (n=37), MCI (n=16) and dementia (n=6) participants of the MiaGB (Microbiome in aging Gut and Brain) consortium. We detected significantly reduced abundance of oral commensals like B. animalis, E. brachy, and F. fastidiosum in MCI and dementia samples than controls with specific increase of P. histicola in MCI and neuronal Aβ1–42 promoting V. parvula in the dementia. In addition, proteome analyses revealed that highly neuronal relevant nitrite-nitrate conversion cycle genes were significantly differed in dementia and MCI than controls. Discriminatory predictive analyses revealed that combination of microbiome and proteome signature are stronger discriminator for dementia/MCI from cognitively healthy controls. Our results indicate the potential links among oral microbiome, proteome and dementia, as well as diagnostic potential for dementia risk.

